# From adhesion complex to signaling hub: the dual role of dystroglycan

**DOI:** 10.3389/fmolb.2023.1325284

**Published:** 2023-12-14

**Authors:** Francesca Sciandra, Manuela Bozzi, Maria Giulia Bigotti

**Affiliations:** ^1^ Istituto di Scienze e Tecnologie Chimiche “Giulio Natta”-SCITEC (CNR), Roma, Italy; ^2^ Dipartimento di Scienze Biotecnologiche di Base, Cliniche Intensivologiche e Perioperatorie, Sezione di Biochimica, Università Cattolica del Sacro Cuore di Roma, Roma, Italy; ^3^ School of Biochemistry, University of Bristol, Bristol, United Kingdom; ^4^ Bristol Heart Institute, Research Floor Level 7, Bristol Royal Infirmary, Bristol, United Kingdom

**Keywords:** dystroglycan, cell adhesion, cytoskeleton, cell signaling, muscular dystrophy

## Abstract

Dystroglycan (DG) is a transmembrane protein widely expressed in multiple cells and tissues. It is formed by two subunits, α− and β-DG, and represents a molecular bridge between the outside and the inside of the cell, which is essential for the mechanical and structural stability of the plasma membrane. The α-subunit is a cell-surface protein that binds to the extracellular matrix (ECM) and is tightly associated with the plasma membrane via a non-covalent interaction with the β-subunit, which, in turn, is a transmembrane protein that binds to the cytoskeletal actin. DG is a versatile molecule acting not only as a mechanical building block but also as a modulator of outside–inside signaling events. The cytoplasmic domain of β-DG interacts with different adaptor and cytoskeletal proteins that function as molecular switches for the transmission of ECM signals inside the cells. These interactions can modulate the involvement of DG in different biological processes, ranging from cell growth and survival to differentiation and proliferation/regeneration. Although the molecular events that characterize signaling through the ECM-DG-cytoskeleton axis are still largely unknown, in recent years, a growing list of evidence has started to fill the gaps in our understanding of the role of DG in signal transduction. This mini-review represents an update of recent developments, uncovering the dual role of DG as an adhesion and signaling molecule that might inspire new ideas for the design of novel therapeutic strategies for pathologies such as muscular dystrophy, cardiomyopathy, and cancer, where the DG signaling hub plays important roles.

## 1 Introduction

Dystroglycan (DG) is a transmembrane protein originally identified in skeletal muscle as part of the dystrophin–glycoprotein complex (DGC), a group of cytoskeletal (dystrophin, syntrophins, and dystrobrevin) and membrane integral (sarcoglycans and sarcospan) proteins (Ibraghimov-Beskrovnaya et al., 1992). DG is also expressed in other tissue districts, including the central and peripheral nervous systems and epithelia (Durbeej et al., 1998). DG constitutes a bridge between the extracellular matrix (ECM) and the cytoskeleton, and its primary function is to structurally stabilize the plasma membrane. This connection is vital for striated muscle, as evidenced by the fact that disrupting the link between the ECM and the interior of the muscle cells causes muscular dystrophies.

**FIGURE 1:**
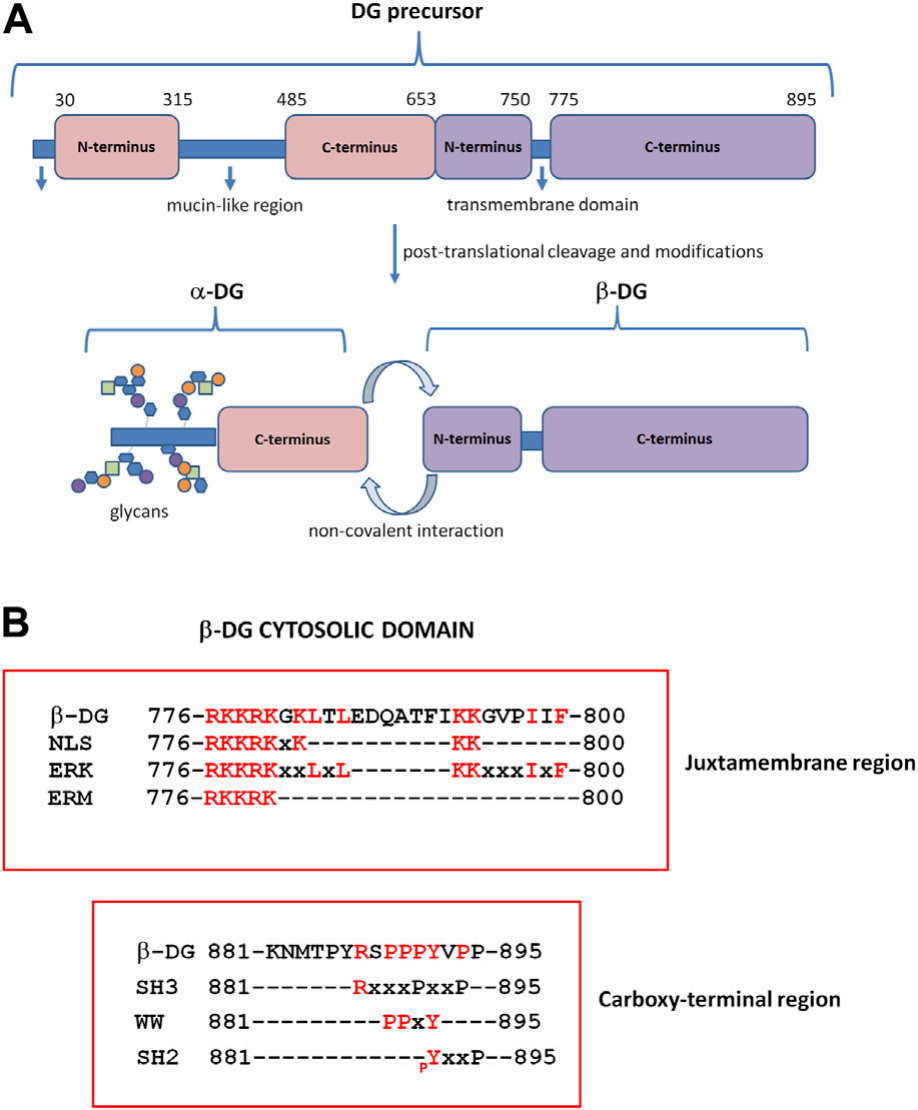
Dystroglycan (DG) domain structure. **(A)** The *DAG1* gene is translated in a precursor polypeptide that is subsequently cleaved to generate the α-DG and β-DG subunits. α-DG consists of a central mucin-like domain and a C-terminal globular domain that is non-covalently linked to β-DG at the cell surface. The N-terminal domain of α-DG is cleaved by furin during the maturation process (Kanagawa et al., 2004). β-DG contains an extracellular N-terminal domain, a transmembrane domain, and a cytoplasmic C-terminus. **(B)** β-DG cytoplasmic domain containing motifs for protein–protein interactions. At the juxtamembrane region, the cytoplasmic domain contains motifs for the interaction with extracellular signal-regulated kinase (ERK), ezrin–radixin–moesin proteins (ERM), and rapsyn; the same region includes a nuclear localization signal (NLS). The C-terminus harbors motifs for binding to WW, SH3, and SH2-containing proteins. pY: specifically phosphorylated tyrosine (modified from Moore and Winder, 2010).

DG is encoded by a single gene (*DAG1*) as a unique precursor that is cleaved into two interacting subunits, α- and β-DG, by a post-translational and autocatalytic process (Ibraghimov-Beskrovnaya et al., 1992) (Figure 1A). α-DG is a peripheral membrane protein that interacts with the ECM laminin globular domain (LG)-containing proteins, such as laminins, agrin, neurexins, and perlecan (Gee et al., 1994; Gesemann et al., 1998; Peng et al., 1999; Sugita et al., 2001). α-DG is characterized by a dumb-bell-like shape formed by two globular domains separated by a highly glycosylated central region (Brancaccio et al., 1995). Glycosylation is extremely heterogeneous and contains a rare phosphorylated O-mannosyl residue linked to a polymer formed by repeated units of the [-3-Xyl-α1, -3GlcA-β1]_n_ disaccharide, known as “matriglycan” (Yoshida-Moriguchi and Campbell, 2015). The majority of ECM-interacting proteins bind directly to these extensive α-DG glycan chains through their LG domains that contain a conserved binding motif characterized by a glycine and a Ca^2+^-binding site surrounded by basic residues (Briggs et al., 2016). The synthesis of matriglycan repeats is finely regulated in time and space (Goddeeris et al., 2013), and α-DG displays diverse glycosylation patterns in different tissues. As a result, α-DG appears as a broad smeared band on Western blotting, with an apparent molecular weight of 156 kDa in skeletal muscle, 140 kDa in cardiac muscle, and 120 kDa in the brain. Moreover, LG-containing proteins, based on specific circumstances, can compete for α-DG binding, thus contributing to the diversity of the ECM along discrete developmental stages and across mammalian tissues (Talts et al., 1999; Kanagawa et al., 2005). When α-DG glycosylation is perturbed and reduced due to mutations in the enzymes that are responsible for the O-mannosyl glycosylation process, muscular dystrophies arise. Collectively known as secondary dystroglycanopathies, these particular types of muscular dystrophies range from mild and late-onset limb-girdle muscular dystrophies to severe congenital muscular dystrophies with and without brain involvement, such as Walker–Warburg syndrome (WWS), muscle–eye–brain disease (MEB), and Fukuyama congenital muscular dystrophy (FCMD) (Kanagawa, 2021).

α-DG retains the contact with the plasma membrane by interacting, non-covalently and independently from its glycosylation state, with the β-subunit, which, in turn, is a transmembrane protein (Sciandra et al., 2001). The N-terminal extracellular domain (or ectodomain) of the β-subunit is intrinsically unfolded (Bozzi et al., 2003), and the α-subunit is its only identified ligand so far (Sciandra et al., 2001). The cytosolic domain of β-DG, like its ectodomain, also has no defined structure, but it is characterized by the presence of several proline residues and different functional motifs for protein–protein interaction (Moore and Winder, 2010) (Figure 1B). The sequence PPxY at the extreme C-terminus is recognized by dystrophin and its analog utrophin (Jung et al., 1995), and through the connection with dystrophin, the DG complex fully establishes the link between the ECM and the cytoskeleton. Caveolin-3, a protein component of the caveolae in the plasma membrane of skeletal, cardiac, and smooth muscles, and the growth factor receptor-bound adaptor protein Grb2 bind to β-DG through the same PPxY motif, thus competing with dystrophin for the binding to DG (Russo et al., 2000; Sotgia et al., 2000). At the intracellular juxtamembrane region, a stretch of basic residues is capable of interacting with ezrin, a protein belonging to the ERM protein family, as well as rapsyn and ERK (Cartaud et al., 1998; Spence et al., 2004) (Figure 1B).

## 2 DG stabilizes diverse protein complexes in multiple tissues

**FIGURE 2:**
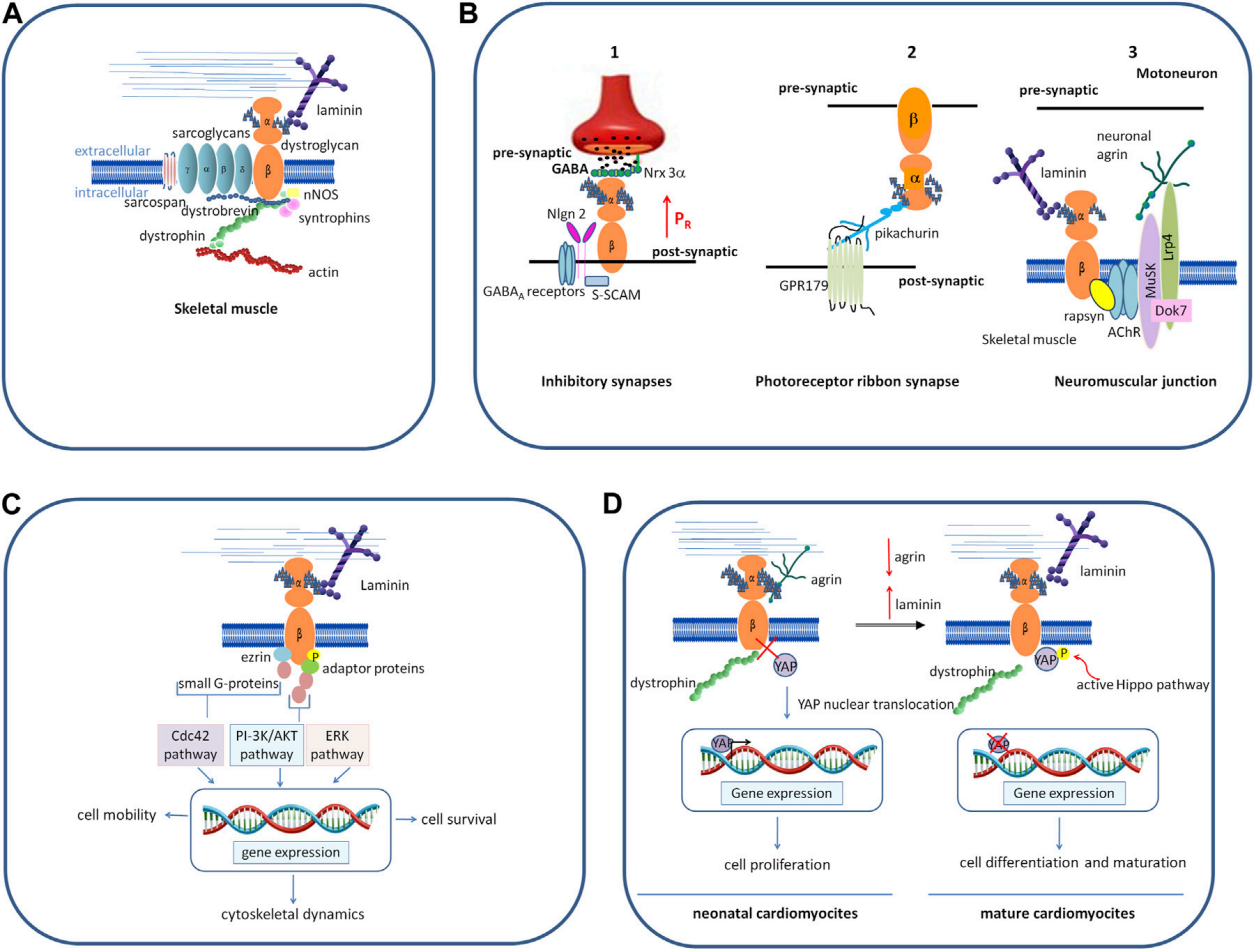
Schematic representation of different roles played by DG. **(A)** In skeletal muscle, as the central component of the DGC, a group of cytoskeletal (dystrophin, syntrophins, and dystrobrevin) and membrane integral proteins (sarcoglycans and sarcospan), DG plays a role as the adhesion molecule anchoring myotubes to the ECM, thus conferring stability to muscle fibers during the continuous cycles of contraction and relaxation. **(B)** In the nervous system, DG organizes and stabilizes the molecular architecture of diverse synapses. 1) In the brain, DG stabilizes the inhibitory synapses of GABA_A_ receptors by interacting with presynaptic adhesion proteins, NRXs, and post-synaptic intracellular protein S-SCAM, which, in turn, interacts with NL2. The binding between DG and NRX-3α enables a normal release probability (the probability of a synaptic vesicle to release its transmitter content in response to an action potential) in the olfactory bulb and the prefrontal cortex (modified from Trotter et al., 2023). 2) In the retina, DG is located pre-synaptically at photoreceptor ribbon synapses and binds the secreted molecule pikachurin, which, in turn, binds to the postsynaptic transmembrane orphan receptor GPR179. 3) At the neuromuscular junction, post-synaptic DG forms a bridge between laminins and the cortical protein rapsyn, and it stabilizes the receptor complex formed by Musk-Dok7-Lrp4 that is needed for the binding to neuronal agrin and the formation of the acetylcholine receptors. **(C)** The interaction of α-DG with laminin induces β-DG phosphorylation, followed by inhibition of its binding with dystrophin. The phosphorylated β-DG cytodomain recruits adaptor protein Grb2 and small G proteins, turning the extracellular information into intracellular signals that ultimately modulate the expression of genes involved in different processes and cell behavior. **(D)** In neonatal cardiomyocytes, α-DG binds to the proteoglycan agrin, and β-DG is tightly associated with dystrophin. YAP reaches the nucleus, activating the expression of genes responsible for cell proliferation. In the adult heart, agrin is downregulated and replaced by laminin (or by another yet unidentified factor) in binding to α-DG, inducing β-DG to loosen its link to dystrophin and sequester phosphorylated YAP out of the nucleus, thus impairing the proliferative program. Abbreviations: NRXs, neurexins; NL2, neuroligin; ERK, extracellular signal-regulated kinase; Musk, muscle specific receptor tyrosine kinase; Dok7, docking-protein 7; Lrp4 low-density lipoprotein receptor-related protein 4; Grb2, growth factor receptor-bound protein-2; PI-3K, phosphatidylinositol 3-kinase; Cdc42, cell division control protein 42 homolog; YAP, yes-associated protein.

In skeletal muscle, as the central part of the DGC DG primarily functions as an adhesion molecule anchoring myotubes to the ECM in order to confer stability to muscle fibers during the continuous cycles of contraction and relaxation (Figure 2A). In addition, the DGC also plays a role in the assembly of the insulin receptors, whose structural integrity and functional properties depend on the bridge that plakoglobin forms between them and the β-DG cytodomain (Eid Mutlak et al., 2020). The development of insulin resistance and insulin-related metabolic disorders, such as hyperinsulinemia and glucose intolerance, often observed in patients affected by muscular dystrophies, may be due to a destabilization of the DGC, which, in turn, leads to a reduced stability and functionality of the insulin receptors.

The correct glycosylation of the α-subunit is crucial for skeletal muscle stability, and its aberrant O-mannosyl glycosylation is associated with the development of secondary dystroglycanopathies (Kanagawa, 2021; Michele et al., 2002). Interestingly, α-DG glycosylation does not appear to exert the same degree of stabilization in all types of tissue districts. For example, in the cardiac muscle of transgenic mice where FKTN, the causative gene of FCMD, is conditionally inactivated, hypo-glycosylation of α-DG leads to cardiac dysfunction only in aged mice despite a marked reduction of the entire DGC (Ujihara et al., 2019). This observation suggests that an unknown compensatory mechanism prevents the young hearts from developing a diseased phenotype and that membrane fragility is not the only pathogenic factor driving disease onset.

At the neuromuscular junction (NMJ), the specialized synapse between motoneurons and skeletal muscle, post-synaptic DG forms a bridge between laminins and the cortical protein rapsyn (Montanaro et al., 1998), and it stabilizes the receptor complex formed by muscle-specific-receptor tyrosine kinase (Musk), docking-protein 7 (Dok7) and low-density lipoprotein receptor-related protein 4 (Lrp4) that is needed for the binding to neuronal agrin. This, in turn, triggers a cascade of events, leading to the clustering of the acetylcholine receptors and the final maturation of the post-synaptic elements (Kim et al., 2008; Guarino et al., 2020) (Figure 2B).

In the central nervous system, DG is expressed in the hippocampus, cerebellum, cerebral cortex, hypothalamus, olfactory bulb, and retina (Zaccaria et al., 2001). Studies carried out in conditional knock-out mice where *DAG1* is genetically deleted in different brain cell types have shown that DG is important for the stability of the molecular architecture of post-synaptic neurons (Moore et al., 2002; Satz et al., 2010; Früh et al., 2016). In the hippocampus and cerebellum, the DG complex stabilizes the inhibitory synapses of GABA_A_ receptors, interacting with presynaptic adhesion proteins, neurexins (NRXs), NRX-like family components (Sugita et al., 2001; Siddiqui and Craig, 2011), and also with post-synaptic intracellular proteins such as S-SCAM, a member of the membrane-associated guanylate kinase (MAGUK) family of PDZ-domain containing proteins. In turn, S-SCAM interacts with the C-terminal tail of neuroligin 2 (NL2), linking the NRX-NL adhesion system with DGC (Sumita et al., 2007; Früh et al., 2016; Briatore et al., 2020; Miller and Wright, 2021). The conditional deletion of the *DAG1* gene in the pyramidal cells of the hippocampus results in a decreased clustering of GABA_A_ receptors and an impairment of synapse transmission (Früh et al., 2016; Miller and Wright, 2021). Recently, Trotter and colleagues, using CRISPR-mediated *DAG1* deletion in mice, showed that the binding between α-DG and NRX-3α is indispensable to activate a trans-synaptic feedback signaling loop that enables a normal release probability (the probability of a synaptic vesicle to release its transmitter content in response to an action potential) at inhibitory synapses in the olfactory bulb and the prefrontal cortex (Trotter et al., 2023). This interaction has no effect on synapse numbers *in vivo*, but it is strictly required for the ability of NRX-3α to organize a fully functional presynaptic release machinery in at least two brain regions (Figure 2B). The nature of the signal activated by DG and transferred to presynaptic terminals is still not known, and hypotheses may include a possible conformational change or dimerization of Nrxn-3α, as well as raising an independent additional signal. Studies aimed at decoding this mechanism will help to understand how mutations in α-DG and the glycosyltransferases dedicated to its decoration with sugars translate into severe neurological defects.

In the retina, DG is located pre-synaptically at photoreceptor ribbon synapses and binds to the secreted LG-domain-containing molecule pikachurin, which, in turn, binds to the postsynaptic transmembrane orphan receptor GPR179 (Sato et al., 2008; Orlandi et al., 2018). Conditional deletion of the *DAG1* gene from photoreceptors*,* as well as hypo-glycosylation of α-DG associated with secondary dystroglycanopathies, results in impaired synaptic transmission (Kanagawa et al., 2010; Hu et al., 2011; Omori et al., 2012) (Figure 2B).

DG also plays an important role in Schwann cell myelination within the central and peripheral nervous systems (Saito et al., 1999; Occhi et al., 2005; Colognato et al., 2007). The interaction between α-DG and laminin-α2 provides an anchorage between the Schwann cell plasma membrane and the ECM, enabling Schwann cells to repeatedly wrap themselves around the axon, thus forming layers of myelin sheath (Masaki and Matsumura, 2010).

As far as the adhesion functions are concerned, it is intriguing that in the central and peripheral nervous systems, α-DG operates independently from the β-subunit as an extracellular scaffold, representing a “landing area” for multiple ECM proteins involved in the guidance of neuron migration and axon extension. Throughout the developing nervous system, α-DG is required for maintaining the proper organization of the ECM by binding to secreted LGs-containing proteins such as laminins and Slit (Wright et al., 2012), and the transmembrane receptor Celsr3, which also contains two LG domains in its large extracellular region (Lindenmaier et al., 2019). These proteins operate as cues that guide axons during the development of neural circuits, and it was demonstrated that α-DG functions autonomously and does not require signaling through the β-DG intracellular domain (Lindenmaier et al., 2019). Moreover, the brain-specific deletion of the cytoplasmic domain of β-DG in conditional knock-out mice had no consequences on brain development; laminin was normally expressed and deposited, and no effects on neuron migration were observed (Satz et al., 2010). Accordingly, in severe forms of secondary dystroglycanopathies, hypo-glycosylated α-DG is unable to maintain the ECM as a permissive substrate for axon growth, and neuro-developmental abnormalities arise, including type II lissencephaly, which is the result of neuronal over-migration (Godfrey et al., 2011).

Much is known about the roles of adhesive proteins and their receptors in the formation of the correct glial scaffold to ensure neuronal migration, but little is understood about the underlying molecular mechanisms. Recently, new progress has been made in the direction of deciphering how the ECM signals are interpreted and integrated within neural cells. It was shown that in radial glial cells, α-DG maintains the proper organization and structure of the basement membrane by binding to β1-integrin through laminin. Downstream of this adhesion complex, Cas, a cytosolic adaptor protein known to participate in integrin adhesion complexes turnover, becomes phosphorylated and stimulates integrin signaling that is indispensable for a correct glial scaffold (Wong et al., 2023).

## 3 DG and signal transduction

As soon as it was identified, DG was described as a molecule acting as a bridge between the ECM and the cytoskeleton, fundamental for the structural stability of the sarcolemma. Subsequently, several *in vitro* biochemical studies demonstrated that the cytosolic domain of the β-subunit is specifically phosphorylated in response to extracellular guidance cues and is capable of binding to several proteins involved in signal transduction. The functions of the DG complex, therefore, were expanded to mechanical sensing and signaling, with DG representing a hub for the signal molecules that were gradually identified.

### 3.1 DG as a mechano-transducing receptor

Cells have the ability to sense the mechanical features of their surrounding micro-environment and respond to any changes, including substrate topography, rigidity, and adhesiveness (Martino et al., 2018). Growing evidence suggests that DG, similarly to integrins, upon changes in its local environment, recruits adaptor proteins, which, in turn, activates small G proteins, thus transforming physical stimuli in cellular responses, such as cytoskeletal remodeling and cell movement, cell survival, proliferation, and differentiation (Figure 2C).

Several studies conducted in myoblasts, myotubes, and some non-muscle cell types have shown that the initial event in the signal cascade is the adhesion-dependent, Src kinase-driven phosphorylation of Tyr890 within the cytodomain of β-DG (James et al., 2000; Ilsley et al., 2001; Sotgia et al., 2001) that abolishes the interaction with utrophin and dystrophin, and leads to the recruitment of Grb2. Grb2 binds to RhoA and Rac1, two small G proteins that act as molecular switches transducing cell survival signals from the outside to the inside of cells (Chardin et al., 1995; Chockalingam et al., 2002; Oak et al., 2003) (Figure 2C). In fibroblasts, in response to cell adhesion, β-DG recruits the cytoplasmic peripheral protein ezrin, belonging to the ERM family, as well as the Rho GTPase protein Dbl, to its proximal membrane sites, driving the activation of Cdc42 and the formation of filopodia (Spence et al., 2004; Batchelor et al., 2007). These latter are actin-rich structures that perceive stimuli from the local microenvironment and transmit them inside the cell during cell adhesion, cell migration, and cell morphogenesis (Chen et al., 2003).

Other notable examples of cell types are i) myotubes, where DG is also involved in mechano-sensing via the phosphoinositide 3-kinase (PI3K)/protein kinase B (AKT) pathway that promotes metabolism and cell survival, and ii) epithelial lung cells, where the binding of DG to laminin culminates with the activation of the ERK1/2 pathway that transduces mechanical stress (Langenbach and Rando, 2002; Takawira et al., 2011) (Figure 2D).

It remains to be clarified how the outside signals received by α-DG are transmitted to β-DG and culminate with the phosphorylation of its cytodomain. The two DG subunits are cleaved at a conserved so-called SEA module, as first identified in a sperm protein, enterokinase, and agrin (Esapa et al., 2003; Wreschner et al., 2009). According to the hypothesis that Wreschner and colleagues formulated for the SEA module-containing proteins, α- and β-DG may remain associated to form a receptor–ligand alliance to regulate signal transduction: glycosylated α-DG bound to its ligands may induce a conformational change in the membrane-associated β-DG and, thus, modulate signal transmission inside the cells. It is also possible that α-DG dissociates from the β-subunit and exerts its functions independently, as already described in the previous section.

Interestingly, although phosphorylation of Tyr890 within the cytodomain of β-DG is crucial for the transmission of cell survival and proliferation signals, its inhibition in the mdx mouse, the animal model for Duchenne muscular dystrophy, improves the dystrophic phenotype by stabilizing the entire DGC without disrupting cell survival signals, highlighting how those may be transduced through alternative pathways via integrins (Miller et al., 2012).

Physiologically, DG-mediated mechano-signaling is highly regulated (Ilsley et al., 2001) and may be perturbed when laminin signaling through the DG complex is attenuated. This is, for example, the case in dystroglycanopathies, where the correct glycosylation of α-DG, responsible for the stability of the laminin/α-DG complex, is severely compromised, although the underlying mechanisms are not fully understood. For example, in epithelial cells transfected with a DG mutant associated with hypoglycosylation of α-DG and with the development of limb girdle muscular dystrophy, β-DG reduces its membrane dynamics and clustering within the actin-rich domains, influencing cell migration and spontaneous cell movement (Palmieri et al., 2017).

### 3.2 Mechano-sensing through the agrin–DG–YAP axis: a new hope for cardiac muscle regeneration

It has been shown in different tissues that DG is involved in the Hippo pathway, a conserved kinase cascade that inhibits cell proliferation. One of the most striking effects of this was found in the cardiac muscle, where DG has been shown to promote or inhibit cardiomyocyte proliferation, following the interaction with different ligands in the ECM. Specifically, in the mice neonatal heart, binding of α-DG to the ECM proteoglycan agrin would keep the β-DG cytodomain preferentially bound to dystrophin, resulting in the co-transcription factor YAP (yes-associated protein) being able to serve its role in the nucleus, where it promotes cell growth and proliferation. In the adult mammalian heart, agrin expression is downregulated and the protein is replaced by another yet unidentified factor in binding to α-DG, leading β-DG to bind and sequester phosphorylated YAP out of the nucleus, thus preventing it from exerting its pro-proliferative activity (Bassat et al., 2017; Morikawa et al., 2017) (Figure 2D).

The relationship between the DG–agrin axis and a possible agrin-driven re-activation of YAP was first demonstrated in adult cardiomyocytes and *in vivo* in mice, opening important implications for cardiac regenerative medicine (Bigotti et al., 2020), especially promising after the recent finding that agrin is expressed in an age-dependent manner also in human cardiac muscle (Skeffington et al., 2022).

Interestingly, the Hippo pathway circuit through DG is also implicated in the maintenance of quiescence of dormant tumor cells in the brain. It was recently demonstrated that laminin-211 deposited by astrocytes induces DG to sequester YAP, thus inhibiting growth-promoting programs that are critical for brain metastasis (Dai et al., 2022). YAP has also been shown to positively regulate skeletal muscle mass and protein synthesis, following injury and atrophy (Watt et al., 2015), and it has differential expression in healthy and dystrophic muscles (Vita et al., 2018; Iyer et al., 2019). Although the functional link between DG and the Hippo signaling pathway in maintaining skeletal muscle integrity still remains unclear, components of such a pathway were recently found to interact with DG in adult fast-twitch oxidative muscles of *Drosophila,* suggesting that the role of DG as a mechano-sensing receptor is evolutionarily conserved in muscle cells (Yatsenko et al., 2020). The importance of studies on insect muscle for uncovering possible roles of DG in signaling has been further underlined by a novel transcriptome analysis of DG-null *Drosophila*, which has shown how DG, together with the DGC, may be part of signaling pathways activated by temperature and metabolic stress (Carney et al., 2023).

### 3.3 β-DG in the nucleus: does it have a signaling function?

β-DG contains a nuclear localization signal (NLS) in its cytoplasmic juxtamembrane region (Oppizzi et al., 2008). In specific pathological conditions, upregulation of metalloproteinases and γ-secretases generates fragments of β-DG that are targeted to the nucleus via an importin-dependent pathway (Lara-Chacó et al., 2010). However, the function and consequences of this nuclear translocation are not yet known. It has been demonstrated that β-DG contributes to the stability of the nuclear membrane in murine myoblasts (Gómez-Monsivais et al., 2020), but it has also been highlighted that the translocation of β-DG fragments into the nucleus is associated with alterations in the expression levels of some genes in prostate cancer cells (Mathew et al., 2013). It remains to be clarified if and how nuclear β-DG exerts control over gene expression and whether this is the consequence of an activation of a regulated cascade of signals leading from the external stimuli to the cell response.

BOX 1 **Open questions on DG and signaling**

a. How does α-DG transmit signals from the ECM to the β-subunit? Does α-DG signaling directly induce a conformational change in β-DG? Or are there other factors involved that elicit and/or mediate signal transduction?
b. How does the glycosylation state of α-DG modulate its binding to ECM? Is there a fine-tuned process of extension of matriglycan that modulates the affinity of α-DG for its ligands? Or does binding to α-DG depend mainly on the concentration/composition of ECM proteins in different physiological or pathological conditions?
c. How does α-DG localize at the cell periphery when it is not coupled to the β-subunit? Are there other specific membrane receptors for α-DG?
d. Does nuclear β-DG play a role in gene expression?

## 4 Conclusion

Years of research on the DG complex have established that this protein is characterized by a remarkable functional versatility. DG is not only an adhesion complex that anchors the sarcolemma of the skeletal muscle to the ECM, stabilizing it during the contraction and relaxation cycles, but also a molecule that senses signals from the ECM and transmits them to the cell, promoting survival, cytoskeleton remodeling, and synapse functions.

Key questions about the molecular mechanisms that drive signaling through the DG complex still remain unsolved (Box 1). The glycosylation of α-DG and the interaction between the two subunits are fundamental for signaling roles as α-DG transmits the ECM signals to the intracellular domain of the β-subunit. Such a mechanism presupposes a modulation of the ability of α-DG to bind to the ECM, as well as a regulation of the cross-talk between α- and β-DG. The ability of α-DG to interact with multiple ECM LG-containing proteins depends on its glycosylation, which is different in different tissues. How these differences in glycosylation patterns arise remains unclear, but the observation that the length of the glycan chains correlates with the binding capacity suggests a fine-tuned extension of glycans that is likely to have important functional consequences (Lindenmaier et al., 2019).

The two subunits also work independently, but it is not yet clear in response to which stimuli they separate. α-DG binds and organizes the ECM to guide neuron migration and axon extension. But how does isolated α-DG remain bound to the cell surface? Could there be another, yet unidentified, α-DG receptor at the plasma membrane? The recent observation that α-DG organizes and stabilizes the developing brain cortex basement membrane through β1-integrin signaling opens a new scenario characterized by a possible cross-talk between the two main ECM receptors (Wong et al., 2023).

Research has revealed several pieces of the DG puzzle, and the effort over the coming years will be to recompose them to gain a complete insight into the mechanisms that regulate the roles of DG in cell signaling. Such fundamental understanding may provide promising opportunities for the design of novel therapeutic strategies to cure muscular dystrophy, cardiomyopathy, and cancer.

## References

[B1] Bassat E. Mutlak Y. E. Genzelinakh A. Shadrin I. Y. Baruch Umansky K. Yifa O. (2017). The extracellular matrix protein agrin promotes heart regeneration in mice. Nature 547, 179–184. 10.1038/nature22978 28581497 PMC5769930

[B2] Batchelor C. L. Higginson J. R. Chen Y. J. Vanni C. Eva A. Winder S. J. (2007). Recruitment of Dbl by ezrin and dystroglycan drives membrane proximal Cdc42 activation and filopodia formation. Cell. Cycle 6, 353–363. 10.4161/cc.6.3.3819 17297291

[B3] Bigotti M. G. Skeffington K. L. Jones F. P. Caputo M. Brancaccio A. (2020). Agrin-mediated cardiac regeneration: some open questions. Front. Bioeng. Biotechnol. 8, 594. 10.3389/fbioe.2020.00594 32612983 PMC7308530

[B4] Bozzi M. Bianchi M. Sciandra F. Paci M. Giardina B. Brancaccio A. (2003). Structural characterization by NMR of the natively unfolded extracellular domain of beta-dystroglycan: toward the identification of the binding epitope for alpha-dystroglycan. Biochemistry 42, 13717–13724. 10.1021/bi034867w 14622018

[B5] Brancaccio A. Schulthess T. Gesemann M. Engel J. (1995). Electron microscopic evidence for a mucin-like region in chick muscle alpha-dystroglycan. FEBS Lett. 368, 139–142. 10.1016/0014-5793(95)00628-M 7615068

[B6] Briatore F. Pregno G. Di Angelantonio S. Frola E. De Stefano M. E. Vaillend C. (2020). Dystroglycan mediates clustering of essential GABAergic components in cerebellar purkinje cells. Front. Mol. Neurosci. 13, 164. 10.3389/fnmol.2020.00164 32982691 PMC7485281

[B7] Briggs D. C. Yoshida-Moriguchi T. Zheng T. Venzke D. Anderson M. E. Strazzulli A. (2016). Structural basis of laminin binding to the LARGE glycans on dystroglycan. Nat. Chem. Biol. 12, 810–814. 10.1038/nchembio.2146 27526028 PMC5030134

[B8] Carney T. D. Hebalkar R. Y. Edeleva E. Çiçek I. Ö. Shcherbata H. R. (2023). Signaling through the dystrophin glycoprotein complex affects the stress-dependent transcriptome in Drosophila. DMM Dis. Model. Mech. 16, dmm049862. 10.1242/dmm.049862 36594281 PMC9922874

[B9] Cartaud A. Coutant S. Petrucci T. C. Cartaud J. (1998). Evidence for *in situ* and *in vitro* association between beta-dystroglycan and the subsynaptic 43K rapsyn protein. Consequence for acetylcholine receptor clustering at the synapse. J. Biol. Chem. 273, 11321–11326. 10.1074/JBC.273.18.11321 9556625

[B10] Chardin P. Cussac D. Maignan S. Ducruix A. (1995). The Grb2 adaptor. FEBS Lett. 369, 47–51. 10.1016/0014-5793(95)00578-W 7641883

[B11] Chen Y. J. Spence H. J. Cameron J. M. Jess T. Ilsley J. L. Winder S. J. (2003). Direct interaction of β-dystroglycan with F-actin. Biochem. J. 375, 329–337. 10.1042/BJ20030808 12892561 PMC1223702

[B12] Chockalingam P. S. Cholera R. Oak S. A. Zheng Y. Jarrett H. W. Thomason D. B. (2002). Dystrophin-glycoprotein complex and Ras and Rho GTPase signaling are altered in muscle atrophy. Am. J. Physiol. - Cell. Physiol. 283, C500–C511. 10.1152/ajpcell.00529.2001 12107060

[B13] Colognato H. Galvin J. Wang Z. Relucio J. Nguyen T. Harrison D. (2007). Identification of dystroglycan as a second laminin receptor in oligodendrocytes, with a role in myelination. Development 134, 1723–1736. 10.1242/dev.02819 17395644

[B14] Dai J. Cimino P. J. Gouin K. H. Grzelak C. A. Barrett A. Lim A. R. (2022). Astrocytic laminin-211 drives disseminated breast tumor cell dormancy in brain. Nat. Cancer. 3, 25–42. 10.1038/s43018-021-00297-3 35121993 PMC9469899

[B15] Durbeej M. Henry M. D. Ferletta M. Campbell K. P. Ekblom P. (1998). Distribution of dystroglycan in normal adult mouse tissues. J. Histochem. Cytochem. 46, 449–457. 10.1177/002215549804600404 9524190

[B16] Eid Mutlak Y. Aweida D. Volodin A. Ayalon B. Dahan N. Parnis A. (2020). A signaling hub of insulin receptor, dystrophin glycoprotein complex and plakoglobin regulates muscle size. Nat. Commun. 11, 1381. 10.1038/s41467-020-14895-9 32170063 PMC7070008

[B17] Esapa C. T. Bentham G. R. B. Schröder J. E. Kröger S. Blake D. J. (2003). The effects of post-translational processing on dystroglycan synthesis and trafficking. FEBS Lett. 555, 209–216. 10.1016/S0014-5793(03)01230-4 14644417

[B18] Früh S. Romanos J. Panzanelli P. Bürgisser D. Tyagarajan S. K. Campbell K. P. (2016). Neuronal dystroglycan is necessary for formation and maintenance of functional CCK-positive basket cell terminals on pyramidal cells. J. Neurosci. 36, 10296–10313. 10.1523/JNEUROSCI.1823-16.2016 27707967 PMC6705590

[B19] Gee S. H. Montanaro F. Lindenbaum M. H. Carbonetto S. (1994). Dystroglycan-alpha, a dystrophin-associated glycoprotein, is a functional agrin receptor. Cell. 77, 675–686. 10.1016/0092-8674(94)90052-3 8205617

[B20] Gesemann M. Brancaccio A. Schumacher B. Ruegg M. A. (1998). Agrin is a high-affinity binding protein of dystroglycan in non-muscle tissue. J. Biol. Chem. 273, 600–605. 10.1074/jbc.273.1.600 9417121

[B21] Goddeeris M. M. Wu B. Venzke D. Yoshida-Moriguchi T. Saito F. Matsumura K. (2013). LARGE glycans on dystroglycan function as a tunable matrix scaffold to prevent dystrophy. Nature 503, 136–140. 10.1038/nature12605 24132234 PMC3891507

[B22] Godfrey C. Foley A. R. Clement E. Muntoni F. (2011). Dystroglycanopathies: coming into focus. Curr. Opin. Genet. Dev. 21, 278–285. 10.1016/j.gde.2011.02.001 21397493

[B23] Gómez-Monsivais W. L. Monterrubio-Ledezma F. Huerta-Cantillo J. Mondragon-Gonzalez R. Alamillo-Iniesta A. García-Aguirre I. (2020). The molecular basis and biologic significance of the β-dystroglycan-emerin interaction. Int. J. Mol. Sci. 21, 5944. 10.3390/ijms21175944 32824881 PMC7504044

[B24] Guarino S. R. Canciani A. Forneris F. (2020). Dissecting the extracellular complexity of neuromuscular junction organizers. Front. Mol. Biosci. 6, 156. 10.3389/fmolb.2019.00156 31998752 PMC6966886

[B25] Hu H. Li J. Zhang Z. Yu M. (2011). Pikachurin interaction with dystroglycan is diminished by defective O-mannosyl glycosylation in congenital muscular dystrophy models and rescued by LARGE overexpression. Neurosci. Lett. 489, 10–15. 10.1016/j.neulet.2010.11.056 21129441 PMC3018538

[B26] Ibraghimov-Beskrovnaya O. Ervasti J. M. Leveille C. J. Slaughter C. A. Sernett S. W. Campbell K. P. (1992). Primary structure of dystrophin-associated glycoproteins linking dystrophin to the extracellular matrix. Nature 355, 696–702. 10.1038/355696a0 1741056

[B27] Ilsley J. L. Sudol M. Winder S. J. (2001). The interaction of dystrophin with β-dystroglycan is regulated by tyrosine phosphorylation. Cell. Signal. 13, 625–632. 10.1016/S0898-6568(01)00188-7 11495720

[B28] Iyer S. R. Shah S. B. Ward C. W. Stains J. P. Spangenburg E. E. Folker E. S. (2019). Differential YAP nuclear signaling in healthy and dystrophic skeletal muscle. Am. J. Physiol. - Cell. Physiol. 317, C48-C57. 10.1152/ajpcell.00432.2018 30995108 PMC6689751

[B29] James M. Nuttall A. Ilsley J. L. Ottersbach K. Tinsley J. M. Sudol M. (2000). Adhesion-dependent tyrosine phosphorylation of β-dystroglycan regulates its interaction with utrophin. J. Cell. Sci. 113 ( Pt 10), 1717–1726. 10.1242/jcs.113.10.1717 10769203

[B30] Jung D. Yang B. Meyer J. Chamberlain J. S. Campbell K. P. (1995). Identification and characterization of the dystrophin anchoring site on β-dystroglycan. J. Biol. Chem. 270, 27305–27310. 10.1074/jbc.270.45.27305 7592992

[B31] Kanagawa M. (2021). Dystroglycanopathy: from elucidation of molecular and pathological mechanisms to development of treatment methods. Int. J. Mol. Sci. 22, 13162. 10.3390/ijms222313162 34884967 PMC8658603

[B32] Kanagawa M. Michele D. E. Satz J. S. Barresi R. Kusano H. Sasaki T. (2005). Disruptionof perlecan binding and matrix assembly by post-translational or genetic disruption of dystroglycan function. Febs Lett. 579, 4792–4796. 10.1016/j.febslet.2005.07.059 16098969

[B33] Kanagawa M. Omori Y. Sato S. Kobayashi K. Miyagoe-Suzuki Y. Takeda S. (2010). Post-translational maturation of dystroglycan is necessary for pikachurin binding and ribbon synaptic localization. J. Biol. Chem. 285, 31208–31216. 10.1074/jbc.M110.116343 20682766 PMC2951195

[B34] Kanagawa M. Saito F. Kunz S. Yoshida-Moriguchi T. Barresi R. Kobayashi Y. M. (2004). Molecular recognition by LARGE is essential for expression of functional dystroglycan. Cell. 117, 953–964. 10.1016/j.cell.2004.06.003 15210115

[B35] Kim N. Stiegler A. L. Cameron T. O. Hallock P. T. Gomez A. M. Huang J. H. (2008). Lrp4 is a receptor for agrin and forms a complex with MuSK. Cell. 135, 334–342. 10.1016/j.cell.2008.10.002 18848351 PMC2933840

[B36] Langenbach K. J. Rando T. A. (2002). Inhibition of dystroglycan binding to laminin disrupts the PI3K/AKT pathway and survival signaling in muscle cells. Muscle Nerve 26, 644–653. 10.1002/mus.10258 12402286

[B37] Lara-Chacó B. Bermú Dez De Leó M. Leocadio D. Gó Mez P. Fuentes-Mera L. Martínez-Vieyra I. (2010). Characterization of an Importin alpha/beta-recognized nuclear localization signal in beta-dystroglycan. J. Cell. Biochem. 110, 706–717. 10.1002/jcb.22581 20512930

[B38] Lindenmaier L. B. Parmentier N. Guo C. Tissir F. Wright K. M. (2019). Dystroglycan is a scaffold for extracellular axon guidance decisions. Elife 8, e42143. 10.7554/eLife.42143 30758284 PMC6395066

[B39] Martino F. Perestrelo A. R. Vinarský V. Pagliari S. Forte G. (2018). Cellular mechanotransduction: from tension to function. Front. Physiol. 9, 824. 10.3389/fphys.2018.00824 30026699 PMC6041413

[B40] Masaki T. Matsumura K. (2010). Biological role of dystroglycan in schwann cell function and its implications in peripheral nervous system diseases. J. Biomed. Biotechnol. 2010, 740403. 10.1155/2010/740403 20625412 PMC2896880

[B41] Mathew G. Mitchell A. Down J. M. Jacobs L. A. Hamdy F. C. Eaton H. (2013). Nuclear targeting of dystroglycan promotes the expression of androgen regulated transcription factors in prostate cancer. Sci. Rep. 3, 2792. 10.1038/srep02792 24077328 PMC3786294

[B42] Michele D. E. Barresi R. Kanagawa M. Saito F. Cohn R. D. Satz J. S. (2002). Post-translational disruption of dystroglycan-ligand interactions in congenital muscular dystrophies. Nature 418, 417–422. 10.1038/nature00837 12140558

[B43] Miller D. S. Wright K. M. (2021). Neuronal Dystroglycan regulates postnatal development of CCK/cannabinoid receptor-1 interneurons. Neural Dev. 16, 4. 10.1186/s13064-021-00153-1 34362433 PMC8349015

[B44] Miller G. Moore C. J. Terry R. La riviere T. Mitchell A. Piggott R. (2012). Preventing phosphorylation of dystroglycan ameliorates the dystrophic phenotype in mdx mouse. Hum. Mol. Genet. 21, 4508–4520. 10.1093/hmg/dds293 22810924 PMC5886373

[B45] Montanaro F. Gee S. H. Jacobson C. Lindenbaum M. H. Froehner S. C. Carbonetto S. (1998). Laminin and alpha-dystroglycan mediate acetylcholine receptor aggregation via a MuSK-independent pathway. J. Neurosci. 18, 1250–1260. 10.1523/jneurosci.18-04-01250.1998 9454835 PMC6792747

[B46] Moore C. J. Winder S. J. (2010). Dystroglycan versatility in cell adhesion: a tale of multiple motifs. Cell. Commun. Signal 8, 3. 10.1186/1478-811X-8-3 20163697 PMC2834674

[B47] Moore S. A. Saito F. Chen J. Michele D. E. Henry M. D. Messing A. (2002). Deletion of brain dystroglycan recapitulates aspects of congenital muscular dystrophy. Nature 418, 422–425. 10.1038/nature00838 12140559

[B48] Morikawa Y. Heallen T. Leach J. Xiao Y. Martin J. F. (2017). Dystrophin-glycoprotein complex sequesters Yap to inhibit cardiomyocyte proliferation. Nature 547, 227–231. 10.1038/nature22979 28581498 PMC5528853

[B49] Oak S. A. Zhou Y. W. Jarrett H. W. (2003). Skeletal muscle signaling pathway through the dystrophin glycoprotein complex and Rac1. J. Biol. Chem. 278, 39287–39295. 10.1074/jbc.M305551200 12885773

[B50] Occhi S. Zambroni D. Carro U. D. Amadio S. Sirkowski E. E. Scherer S. S. (2005). Development/plasticity/repair both laminin and schwann cell dystroglycan are necessary for proper clustering of sodium channels at nodes of ranvier. 10.1523/JNEUROSCI.2068-05.2005 PMC1409814 16221851

[B51] Omori Y. Araki F. Chaya T. Kajimura N. Irie S. Terada K. (2012). Presynaptic dystroglycan-pikachurin complex regulates the proper synaptic connection between retinal photoreceptor and bipolar cells. J. Neurosci. 32, 6126–6137. 10.1523/JNEUROSCI.0322-12.2012 22553019 PMC6622127

[B52] Oppizzi M. L. Akhavan A. Singh M. Fata J. E. Muschler J. L. (2008). Nuclear translocation of b-dystroglycan reveals a distinctive trafficking pattern of autoproteolyzed mucins. Authors J. Compil. 9, 2063–2072. 10.1111/j.1600-0854.2008.00822.x PMC2950207 18764929

[B53] Orlandi C. Omori Y. Wang Y. Cao Y. Ueno A. Roux M. J. (2018). Transsynaptic binding of orphan receptor GPR179 to dystroglycan-pikachurin complex is essential for the synaptic organization of photoreceptors. Cell. Rep. 25, 130–145. 10.1016/j.celrep.2018.08.068 30282023 PMC6203450

[B54] Palmieri V. Bozzi M. Signorino G. Papi M. De Spirito M. Brancaccio A. (2017). α-Dystroglycan hypoglycosylation affects cell migration by influencing β-dystroglycan membrane clustering and filopodia length: a multiscale confocal microscopy analysis. Biochim. Biophys. Acta - Mol. Basis Dis. 1863, 2182–2191. 10.1016/j.bbadis.2017.05.025 28572004

[B55] Peng H. B. Xie H. Rossi S. G. Rotundo R. L. (1999). Acetylcholinesterase clustering at the neuromuscular junction involves perlecan and dystroglycan. J. Cell. Biol. 145, 911–921. 10.1083/jcb.145.4.911 10330416 PMC2133180

[B56] Russo K. Di Stasio E. Macchia G. Rosa G. Brancaccio A. Corinna Petrucci T. (2000). Characterization of the β-dystroglycan-growth factor receptor 2 (Grb2) interaction. Biochem. Biophys. Res. Commun. 274, 93–98. 10.1006/bbrc.2000.3103 10903901

[B57] Saito F. Masaki T. Kamakura K. Anderson L. V. B. Fujita S. Fukuta-Ohi H. (1999). Characterization of the transmembrane molecular architecture of the dystroglycan complex in schwann cells. J. Bio. Chem. 274, 8240-8246. 10.1074/jbc.274.12.8240 10075729

[B58] Sato S. Omori Y. Katoh K. Kondo M. Kanagawa M. Miyata K. (2008). Pikachurin, a dystroglycan ligand, is essential for photoreceptor ribbon synapse formation. Nat. Neurosci. 11, 923–931. 10.1038/nn.2160 18641643

[B59] Satz J. S. Ostendorf A. P. Hou S. Turner A. Kusano H. Lee J. C. (2010). Distinct functions of glial and neuronal dystroglycan in the developing and adult mouse brain. J. Neurosci. 30, 14560–14572. 10.1523/JNEUROSCI.3247-10.2010 20980614 PMC2979314

[B60] Sciandra F. Schneider M. Giardina B. Baumgartner S. Petrucci T. C. Brancaccio A. (2001). Identification of the beta-dystroglycan binding epitope within the C-terminal region of alpha-dystroglycan. Eur. J. Biochem. 268, 4590–4597. 10.1046/j.1432-1327.2001.02386.x 11502221

[B61] Siddiqui T. J. Craig A. M. (2011). Synaptic organizing complexes. Curr. Opin. Neurobiol. 21, 132–143. 10.1016/j.conb.2010.08.016 20832286 PMC3016466

[B62] Skeffington K. L. Jones F. P. Suleiman M. S. Caputo M. Brancaccio A. Bigotti M. G. (2022). Determination of agrin and related proteins levels as a function of age in human hearts. Front. Cardiovasc. Med. 9, 813904. 10.3389/fcvm.2022.813904 35355976 PMC8959542

[B63] Sotgia F. Lee H. Bedford M. T. Petrucci T. C. Sudol M. Lisanti M. P. (2001). Tyrosine phosphorylation of-dystroglycan at its WW domain binding motif, PPxY, recruits SH2 domain containing proteins. 10.1021/bi011247r 11724572

[B64] Sotgia F. Lee J. K. Das K. Bedford M. Petrucci T. C. Macioce P. (2000). Caveolin-3 directly interacts with the C-terminal tail of β-dystroglycan. Identification of a central WW-like domain within caveolin family members. J. Biol. Chem. 275, 38048–38058. 10.1074/jbc.M005321200 10988290

[B65] Spence H. J. Chen Y. J. Batchelor C. L. Higginson J. R. Suila H. Carpen O. (2004). Ezrin-dependent regulation of the actin cytoskeleton by β -dystroglycan. Hum. Mol. Genet. 13, 1657–1668. 10.1093/hmg/ddh170 15175275

[B66] Sugita S. Saito F. Tang J. Satz J. Campbell K. Südhof T. C. (2001). A stoichiometric complex of neurexins and dystroglycan in brain. J. Cell. Biol. 154, 435–445. 10.1083/jcb.200105003 11470830 PMC2150755

[B67] Sumita K. Sato Y. Iida J. Kawata A. Hamano M. Hirabayashi S. (2007). Synaptic scaffolding molecule (S-SCAM) membrane-associated guanylate kinase with inverted organization (MAGI)-2 is associated with cell adhesion molecules at inhibitory synapses in rat hippocampal neurons. J. Neurochem. 100, 154–166. 10.1111/j.1471-4159.2006.04170.x 17059560

[B68] Takawira D. Budinger G. R. S. Hopkinson S. B. Jones J. C. R. (2011). A dystroglycan/plectin scaffold mediates mechanical pathway bifurcation in lung epithelial cells. J. Biol. Chem. 286, 6301–6310. 10.1074/jbc.M110.178988 21149456 PMC3057847

[B69] Talts J. F. Andac Z. Göhring W. Brancaccio A. Timpl R. (1999). Binding of the G domains of laminin alpha1 and alpha2 chains and perlecan to heparin, sulfatides, alpha-dystroglycan and several extracellular matrix proteins. EMBO J. 18, 863–870. 10.1093/emboj/18.4.863 10022829 PMC1171179

[B70] Trotter J. H. Wang C. Y. Zhou P. Nakahara G. Sudhof T. C. (2023). A combinatorial code of neurexin-3 alternative splicing controls inhibitory synapses via a trans-synaptic dystroglycan signaling loop. Nature Communications. 10.1038/s41467-023-36872-8 PMC10063607 36997523

[B71] Ujihara Y. Kanagawa M. Mohri S. Takatsu S. Kobayashi K. Toda T. (2019). Elimination of fukutin reveals cellular and molecular pathomechanisms in muscular dystrophy-associated heart failure. Nat. Commun. 10, 5754. 10.1038/s41467-019-13623-2 31848331 PMC6917736

[B72] Vita G. L. Polito F. Oteri R. Arrigo R. Ciranni A. M. Musumeci O. (2018). Hippo signaling pathway is altered in Duchenne muscular dystrophy. PLoS One 13, e0205514. 10.1371/journal.pone.0205514 30304034 PMC6179272

[B73] Watt K. I. Turner B. J. Hagg A. Zhang X. Davey J. R. Qian H. (2015). The Hippo pathway effector YAP is a critical regulator of skeletal muscle fibre size. Nat. Commun. 6, 6048. 10.1038/ncomms7048 25581281

[B74] Wong W. Estep J. A. Treptow A. M. Rajabli N. Jahncke J. N. Ubina T. (2023). An adhesion signaling axis involving Dystroglycan, β1-Integrin, and Cas adaptor proteins regulates the establishment of the cortical glial scaffold. PLoS Biol. 21, e3002212. 10.1371/journal.pbio.3002212 37540708 PMC10431685

[B75] Wreschner D. H. McGuckin M. A. Williams S. J. Baruch A. Yoeli M. Ziv R. (2009). Generation of ligand-receptor alliances by “SEA” module-mediated cleavage of membrane-associated mucin proteins. Protein Sci. 11, 698–706. 10.1110/ps.16502 PMC2373471 11847293

[B76] Wright K. M. Lyon K. A. Leung H. Leahy D. J. Ma L. Ginty D. D. (2012). Dystroglycan organizes axon guidance cue localization and axonal pathfinding. Neuron 76, 931–944. 10.1016/j.neuron.2012.10.009 23217742 PMC3526105

[B79] Yatsenko A. S. Kucherenko M. M. Xie Y. Aweida D. Urlaub H. Scheibe R. J. (2020). Profiling of the muscle-specific dystroglycan interactome reveals the role of Hippo signaling in muscular dystrophy and age-dependent muscle atrophy. BMC medicine 18 (1), 8. 10.1186/s12916-019-1478-3 31959160 PMC6971923

[B77] Yoshida-Moriguchi T. Campbell K. P. (2015). Matriglycan: a novel polysaccharide that links dystroglycan to the basement membrane. Glycobiology 25, 702–713. 10.1093/glycob/cwv021 25882296 PMC4453867

[B78] Zaccaria M. L. Di Tommaso F. Brancaccio A. Paggi P. Petrucci T. C. (2001). Dystroglycan distribution in adult mouse brain: a light and electron microscopy study. Neuroscience 104, 311–324. 10.1016/S0306-4522(01)00092-6 11377836

