# Corrigendum: From adhesion complex to signaling hub: the dual role of dystroglycan

**DOI:** 10.3389/fmolb.2024.1388846

**Published:** 2024-03-18

**Authors:** Francesca Sciandra, Manuela Bozzi, Maria Giulia Bigotti

**Affiliations:** ^1^ Istituto di Scienze e Tecnologie Chimiche “Giulio Natta”-SCITEC (CNR), Rome, Italy; ^2^ Dipartimento di Scienze Biotecnologiche di Base, Cliniche Intensivologiche e Perioperatorie, Sezione di Biochimica, Università Cattolica del Sacro Cuore di Roma, Rome, Italy; ^3^ School of Biochemistry, University of Bristol, Bristol, United Kingdom; ^4^ Bristol Heart Institute, Research Floor Level 7, Bristol Royal Infirmary, Bristol, United Kingdom

**Keywords:** dystroglycan, cell adhesion, cytoskeleton, cell signaling, muscular dystrophy

In the published article, there was an error in the **Funding** statement. The **Funding** statement for the Funder Wellcome Trust was displayed as “Wellcome Trust Institutional Translation Partnership Award, University of Bristol, 2023 (MGB)” but missed the Grant number. The correct **Funding** statement appears below.

